# Observation of plasma inflows in laser-produced Sn plasma and their contribution to extreme-ultraviolet light output enhancement

**DOI:** 10.1038/s41598-023-28500-8

**Published:** 2023-02-01

**Authors:** Kentaro Tomita, Yiming Pan, Atsushi Sunahara, Kouichiro Kouge, Hakaru Mizoguchi, Katsunobu Nishihara

**Affiliations:** 1 Division of Quantum Science and Engineering, Graduate School of Engineering, Hokkaido University, Kita 13, Nishi 8, Kita-Ku, Sapporo, Hokkaido 060-8628 Japan; 2 Interdisciplinary Graduate School of Engineering Sciences, Kyushu University, Kasuga, Fukuoka 816-8580 Japan; 3 Center for Materials Under eXtreme Environment (CMUXE), School of Nuclear Engineering, Purdue University, 500 Central Drive, West Lafayette, IN 47907 USA; 4 Institute of Laser Engineering, Osaka University, 2-6 Yamadaoka, Suita, Osaka 565-0871 Japan; 5 Gigaphoton Inc., 400 Yokokurashinden, Oyama-Shi, Tochigi 323-8558 Japan; 6 Present Address: 24-8-406 Tatsuno-Cho, Hiratsuka, Kanagawa 254-0046 Japan

**Keywords:** Laser-produced plasmas, Lasers, LEDs and light sources, Electrical and electronic engineering, Engineering

## Abstract

Plasma dynamics are governed by electron density (*n*_e_), electron temperature (*T*_e_), and radiative energy transfer as well as by macroscopic flows. However, plasma flow-velocity fields (*v*_flow_) inside laser-produced plasmas (LPPs) have rarely been measured, owing to their small sizes (< 1 mm) and short lifetimes (< 100 ns). Herein, we report, for the first time, two-dimensional (2D) ***v***_flow_ measurements of Sn-LPPs (“double-pulse” scheme with a CO_2_ laser) for extreme-ultraviolet (EUV) light sources for semiconductor lithography using the collective Thomson scattering technique, which is typically used to measure *n*_e_, *T*_e_, and averaged ionic charge (*Z*) of plasmas. Inside the EUV source, we observed plasma inflow speed exceeding 10^4^ m/s magnitudes toward a plasma central axis from its peripheral regions. The time-resolved 2D profiles of *n*_e_, *T*_e_, *Z*, and ***v***_flow_ indicate that the plasma inflows maintain the EUV source at a temperature suitable (25 eV < *T*_e_ < 40 eV) for EUV light emission at a high density (*n*_e_ > 3 × 10^24^ m^−3^) and for a relatively long time (> 10 ns), resulting increment of total EUV light emission. These results indicate that controlling the plasma flow can improve EUV light output and that there is potential to increase the EUV output further.

## Introduction

A lithography light source with a short wavelength is required for fine processing in the semiconductor manufacturing process that supports the IT society, and extreme-ultraviolet (EUV) light with a wavelength of 13.5 nm from laser-generated tin (Sn) plasma is currently used^1–7^. The optical system for EUV lithography has only a reflective optical system, and even if a Mo/Si multilayer mirror with high reflectance of 0.67 is used, a very high light source output is required because there are 12 reflection mirrors in a present EUV lithography tool^8^.

High-density plasma is desired to obtain high output, however, self-absorption cannot be ignored when the density is too high. Therefore, it is necessary to maintain plasma of appropriate density for a relatively long time. It has been clarified that a “double-pulse method” is effective to generate EUV sources with high conversion efficiency (CE) of converting drive laser light into usable in-band EUV photons^5^. In this method, a small (20–30 µm diameter) tin droplet is irradiated with a pre-pulse laser and a main laser pulse for generating a light source plasma. Various papers have already pointed out that the double-pulse method is effective in improving CE^3,9–11^. Further improvements in the efficiency of light sources have been considered to replace the CO_2_ laser as the main pulse with a 2 μm wavelength solid-state laser having high electrical-to-optical conversion efficiency^12–16^. Therefore, it is meaningful to understand the detailed mechanism of how the double-pulse method can provide higher conversion efficiency. One of the crucial problems is the difficulties to measure plasma fundamental parameters (electron density, electron temperature, and charge state *Z*) inside a very small (< 1 mm), non-uniform, short lived (< 100 ns) and transient EUV sources. These fundamental parameters are crucial in increasing in-band EUV (wavelength λ = 13.5 nm, 2% full-bandwidth) output, as pointed out by atomic modeling studies^7,17,18^. They indicate that the EUV source should be in adequate electron density (*n*_e_: 3 × 10^24^–10^25^ m^−3^) and electron temperature (*T*_e_: 25–40 eV) to realize optimum charge state of 8^+^–12^+^.

One (and probably an only) example of time-resolved 2D profiles of *n*_e_, *T*_e_ and averaged charge state ($\overline{Z}$*)* is our previous study, in which the ion term spectra of collective Thomson scattering (CTS) were measured using a custom-built spectrometer^19^. In our previous study, the EUV sources were generated with the double-pulse method, in which a pico-second-pulse Nd:YVO_4_ laser with wavelength of 1064 nm was used as a pre-pulse laser and a carbon dioxide (CO_2_) laser with 15 ns pulse width and wavelength of 10.6 µm were used as the main laser. The CTS results clarified that 2D profiles of *n*_e_ and *T*_e_ significantly changed with a delay time between the pre-pulse laser and the main laser. The CTS results show that the large volume of the optimum plasma conditions was crucial for the high CE.

Here in this paper, for the first time, we clarified 2D-velocity field (***v***_flow_) inside EUV sources. As a result, we firstly discovered the plasma “inflows” toward the main-laser axis (radius *r* = 0) play a key role to increase the CE. It was found that directions of ***v***_flow_ exceeding 10^4^ m/s magnitude was opposite within only a range of 200 µm scale. This unique plasma flows, i.e., the inflows to the direction of *r* = 0, maintain the EUV source at a temperature suitable for EUV light emission for a relatively long time and at a high density. This study firstly shows experimental evidence that controlling the fluid dynamics can be a key technique to improve EUV light output. In addition, the results mention that there still are potentials to increase EUV output power in the future.

This paper is organized as follows. In Results section, we show our experimental setup and 2D profiles of plasma flow velocity field (***v***_flow_) and pressure in the EUV sources. Based on these results, in Discussion section, we discuss how the plasma flows contribute to increasing the total amount of EUV light emission. In Method section, we describe the CTS technique, especially determination processes of ***v***_flow_.

## Results

**Figure 1:**
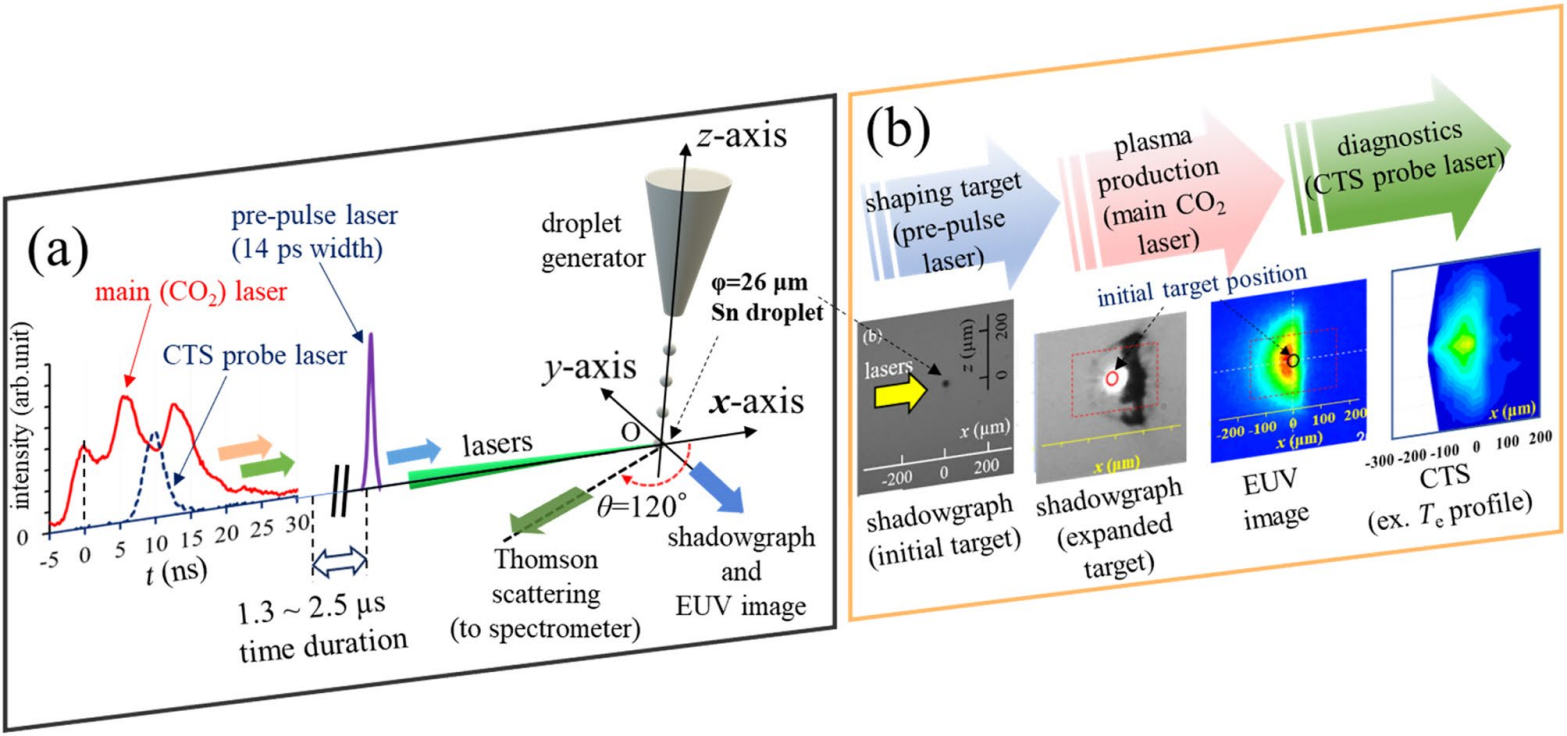
(**a**) Schematic view of the experimental layout and temporal profiles of the Main (CO_2_) and the Probe lasers. (**b**) Examples of shadowgraph (initial target and expanded target), in-band EUV image, and 2D electron temperature profile in perspective view. These results in plan view are shown in Fig. 2 and Supplementary Fig. 3.

Figure 1a schematically shows the experimental setup, which is basically the same configuration as shown in our previous paper^19^. To produce the plasma, first, the Sn droplet target (diameter: 26 μm) was supplied by a droplet generator inside a vacuum chamber (< 10^−4^ Pa). Next, a pre-pulse laser (a Nd:YVO_4_ laser with a 2 mJ energy, a 14 ps pulse, spot diameter 66 µm, wavelength 1064 nm, Gaussian-shaped profile) was used to expand the Sn droplet. In this study, the diameter of the 1/e^2^ intensity was used for the laser spot size. After that, the main laser (a CO_2_ laser with a 100 mJ energy, a 15 ns pulse width, spot diameter 400 µm, wavelength 10.6 µm, Gaussian-shaped profile) was used to produce hot and dense plasmas. By changing the time interval between the pre-pulse laser and the main laser to be 1.3 μs, 2.0 μs, and 2.5 μs, three different plasmas were generated. In this paper, the plasmas are called using the time interval, e.g., “the 2.5 μs-plasma” means the plasma generated with the time interval of 2.5 μs. Measurements of the absolute conversion efficiency (CE) were performed using a calibrated EUV photodetector, which was composed of a spectral purify filter, a narrow-band EUV multilayer mirror, and a photodetector. The in-band EUV radiation (wavelength of λ = 13.5 nm, 2% full-bandwidth) was measured with this device located at an angle of 150° from the positive *x*-axis direction (*θ* = 150°)^1,8^. The CE for a solid angle of 2π sr was calculated assuming an isotropic distribution of the EUV radiation. The 2.0 μs-plasma had the maximum CE herein (4.0%). The CE values were 3.1% and 2.8% for the 1.3 μs- and 2.5 μs- plasmas, respectively. To perform the CTS measurements, the CTS probe laser (a second harmonic of a Nd:YAG laser with 3–10 mJ energy, a 6 ns pulse width, spot diameter of 50 µm, wavelength λ_0_ = 532 nm) was propagated in the positive-*x* direction. As shown in Fig. 1a, all the three lasers (the pre-pulse, the main, and the probe lasers) had identical beam paths.

A fraction of the CTS signals was obtained and focused on an entrance slit of a custom-built spectrometer, which includes 6 reflective gratings and an intensified-CCD (ICCD) camera (Princeton Instruments, PI-MAX4)^19,20^. Because the spatial profiles of the CTS spectra to the *x*-axis direction (the probe-laser beam path) was imaged in a slit height direction, spatially resolved CTS measurements were achieved^21,22^. The relation between the probe-laser beam path (*x*-axis direction) and the slit heigh direction is visually explained in Supplementary Fig. 1a. Regarding the plasma heating by the probe laser, the relative temperature increase (Δ*T*_e_/*T*_e_) was estimated to be less than 3% for the cases reported here. More detailed discussions about the plasma heating by the probe laser are discussed in APPENDIX of ref.^23^.

In Fig. 1a, waveforms of the main laser and the probe lasers are depicted. The time zero (*t* = 0 ns) was defined as the time of the first peak of the main laser. The CTS measurements were performed at times of *t* = 5, 10, 15, and 20 ns and at 0, 50, 100, 150, 200, and 300 μm in the *y-*axis (radial) direction. The time resolution was 5 ns. Sufficient symmetry of the plasma along the *y*-axis (radial) was confirmed, as have been discussed in our previous paper^19^. An example of a CTS image was shown in Supplementary Fig. 1. The way to obtain *n*_e_, *T*_e_, $\overline{Z}$, and plasma flow-velocity field (***v***_flow_) from the CTS results are explained in Method section. As shown in Method section, the Doppler shift of the CTS spectra were analyzed to determine ***v***_flow_. Measurements of shadowgraph and EUV imaging were also performed as shown in Fig. 1a and b.

**Figure 2:**
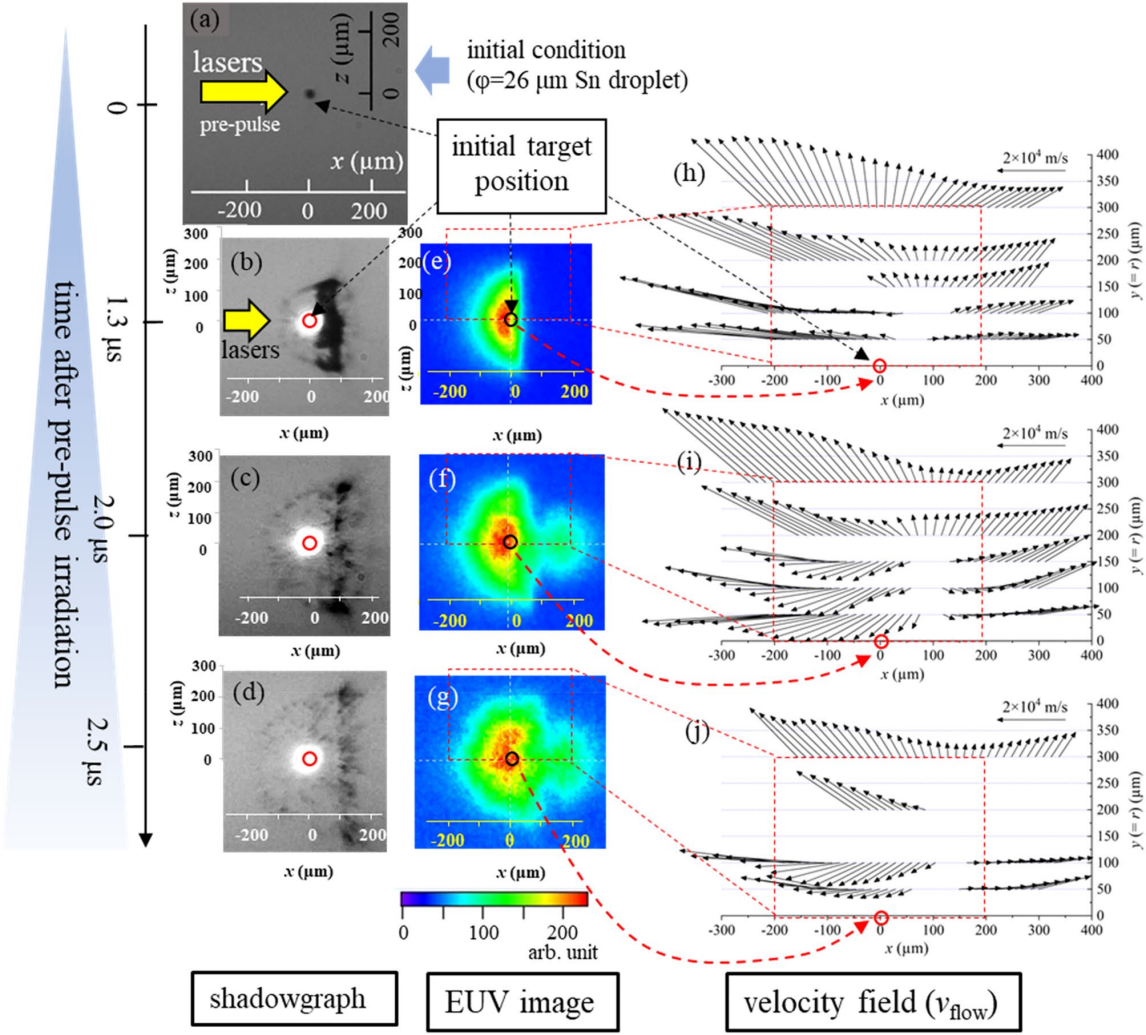
(**a**–**d**) Shadowgraphs of the Sn droplet target before (**a**) and after 1.3 μs (**b**), 2.0 μs (**c**), and 2.5 μs (**d**) irradiating the pre-pulse lasers. (**e**–**g**) Line-integrated in-band EUV images of the 1.3 μs-plasma, the 2.0 μs-plasma, and the 2.5 μs-plasma. (**h**–**j**) Two-dimensional profiles of plasma flow-velocity field (***v***_flow_) of the 1.3 μs-plasma, the 2.0 μs-plasma, and the 2.5 μs-plasma. These ***v***_flow_ profiles were measured at *t* = 10 ns.

**Figure 3:**
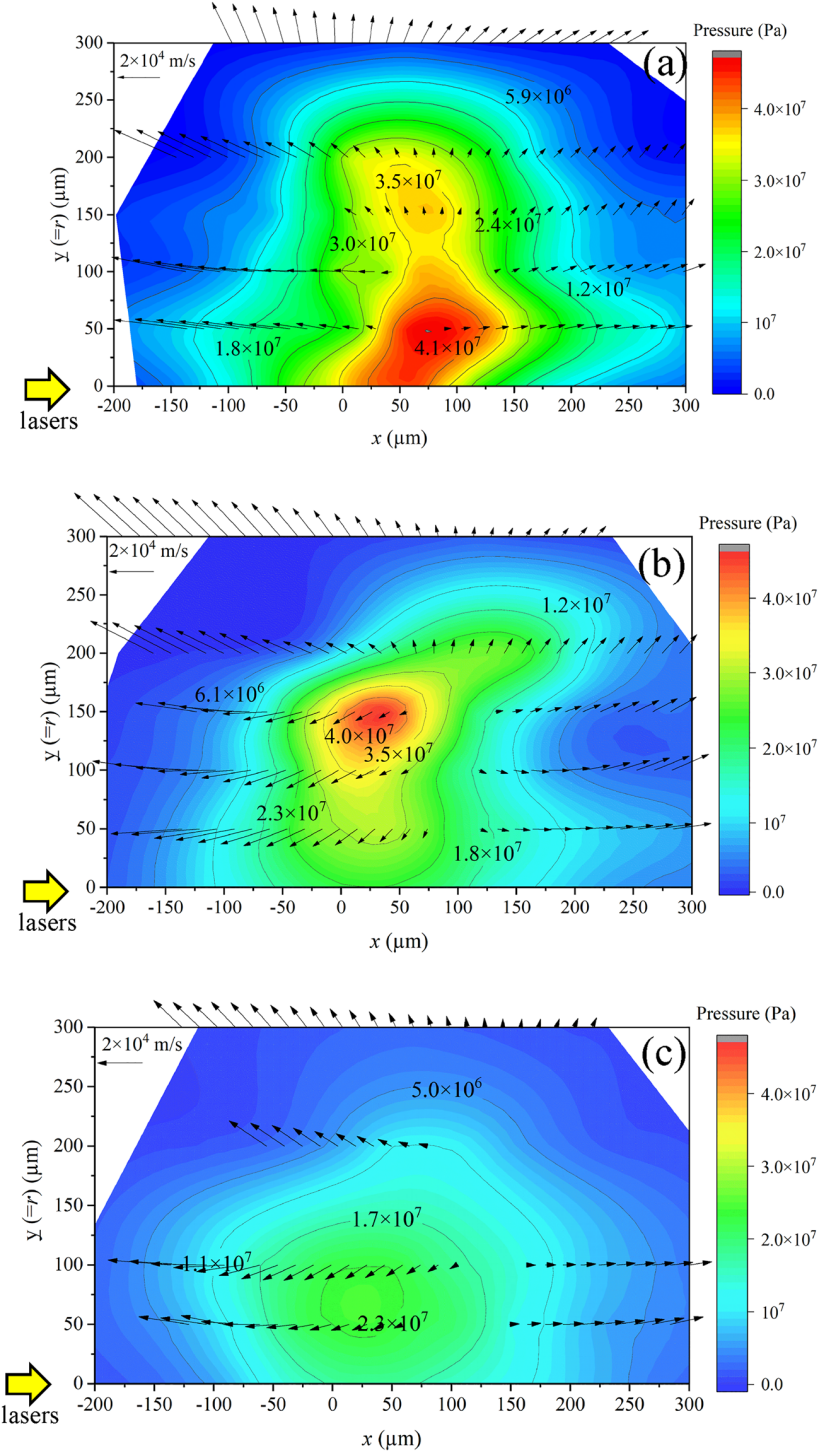
2D-profiles of pressure and plasma flow-velocity field (***v***_flow_) of (**a**) the 1.3 µs-plasma, (**b**) the 2.0 µs-plasma, and (**c**) the 2.5 µs-plasma at a time of *t* = 10 ns.

Here we explain the experimental results. Figure 2a–d show shadowgraphs of the Sn droplet target before [Fig. 2a] and after 1.3 μs [Fig. 2b], 2.0 μs [Fig. 2c], and 2.5 μs [Fig. 2d] irradiating the pre-pulse lasers. Expansion dynamics of a droplet irradiated by pulse laser has been investigated experimentally and theoretically^12,24–30^. 2D radiation hydrodynamics simulation by STAR2d^31^ shows that a high pressure of about 30 GP can be generated on the tin droplet surface at pre-pulse laser condition of 3.7 × 10^12^ W/cm^2^. Convergence of a shock wave driven by the high pressure at the surface to the droplet center and subsequent its divergence result in cavitation in the central region caused by the strong tensile stress (see also Supplementary Fig. 2). The liquid–vapor phase coexistence region is then formed. Reflection of a shock wave from the rear side of a droplet may also cause spallation due to highly stretching, which causes asymmetry as observed in the shadowgraphs in the front and rear sides of the droplet. These shadowgraph images are very much similar to previously observed ones^12,24,25,27,30^. Figure 2e–g show line-integrated in-band EUV images of the three different plasmas, which were measured at the negative *y*-direction. In Fig. 2h–j, the 2D-***v***_flow_ profiles at the positive-*y* region obtained by the CTS spectra measured at *t* = 10 ns are plotted as black arrows. The starting point of each arrows shows the measurement points and the length of the arrows correspond absolute values of ***v***_flow_. The same 2D-***v***_flow_ profiles are also plotted in Fig. 3a–c, in which 2D plasma pressure (*p*) profiles are superimposed as contour plots. The values of *p* were calculated as *p* = *n*_e_*κT*_e_ + *n*_i_*κT*_i_, where *κ* is Boltzmann constant, and *T*_i_ are ion temperature. The 2D profiles of *n*_e_, *n*_i_, and *T*_e_, which are necessary to calculate *p*, are shown in Supplementary Figs. 3 (*T*_i_ = *T*_e_ was assumed in this paper). Note that the vertical axis for Fig. 2a–g (the *z*-axis) are different from the axis for Figs. 2h–j and 3a–c (the *y*-axis). This is because the probe-laser for the CTS measurements was scanned to the *y*-axis direction.

## Discussions

**Figure 4:**
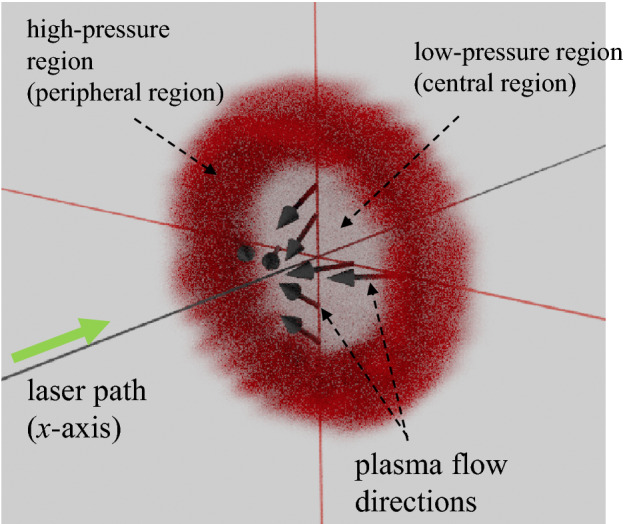
cartoon of the 3D pressure profiles and plasma inflow of the EUV source plasmas.

Here we discuss the CTS results shown in Fig. 3a–c. In these figures, following two points should be emphasized: (i) both the direction and magnitude of ***v***_flow_ varied with positions, (ii) there exist the plasma flows toward the central plasma axis (*x*-axis) as shown in Fig. 3b and c. Figure 4 shows a visualization of the plasma inflow towards the *x*-axis for the case of Fig. 3b. Note that in Fig. 3a–c, only the positive-*y* regions are plotted (the negative-*y* regions are not plotted). Therefore, the bottoms of these graphs show the central axis (the *x*-axis). We further explain these two points in detail based on the 2.0-µs plasma [Fig. 3b], which has the highest CE of 4% in this experiment. Regarding (i), in the region of *x* < 50 µm, the plasma flow is in the negative *x* direction, and in *x* > 100 µm, the flow is in the positive *x* direction. In addition, there is a velocity component perpendicular to the *x*-axis, (i.e., the *y*-axis or the radial direction), although the plasma flow has a large component parallel to the *x*-axis. Regarding (ii), the flow components toward central axis were observed in the region close to the plasma central axis (*y* = *r* < 150 µm). In the region of *y* = *r* > 200 µm, the radial component of ***v***_flow_ were in the direction away from the central axis. Because the magnitude of ***v***_flow_ increased as further away from a specific local region (50 µm < *x* < 100 µm and 100 µm < *y* = *r* < 150 µm), it is expected that plasma flows out from the specific local region to its peripheral regions.

In Fig. 3b, the highest pressure (> 4 × 10^7^ Pa) was formed around the position of (*x*_*p*_,*y*_*p*_) = (30 µm, 150 µm). Generally, pressure gradient can be a main force to generate plasma flows, i.e., the 2D-*v*_flow_ profiles shown in Fig. 3a–c are considered to be formed by pressure gradient forces and plasma flows from higher pressure regions to lower pressure regions. However, the spatial distribution of the direction of the velocity vector shown in Fig. 3b indicates that the plasma is flowing outward from around the position (*x*_*v*_,*y*_*v*_) = (90 µm, 150 µm), which is 60 µm away from the peak pressure position (*x*_*p*_,*y*_*p*_) = (30 µm, 150 µm). This discrepancy is due to the phase difference between acceleration (pressure gradient) and flow velocity, i.e., the difference in definition time. We measured time-resolved peak pressure position at *t* = 5, 15 ns. As a results, we confirmed that the peak pressure position moved from (*x*,*y*) = (70 µm, 150 µm) at *t* = 5 ns to (*x*,*y*) = (10 µm, 150 µm) at *t* = 15 ns. The plasma flows toward the plasma central axis were observed only when the hollow-like pressure structure appeared. For example, there is no plasma inflow for the case of the 1.3 µs-plasma, in which no hollow-like pressure structure was observed [Fig. 3a].

**Figure 5:**
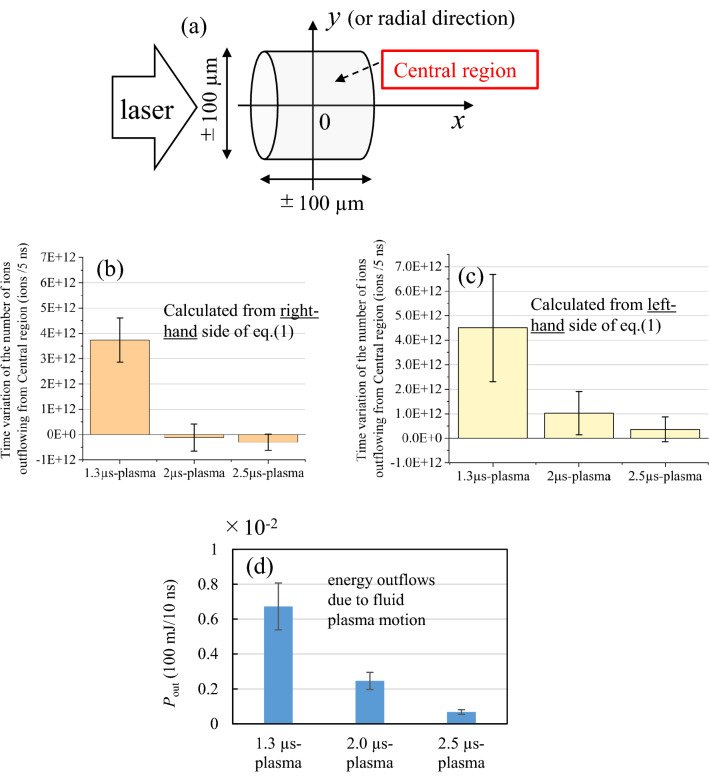
(**a**) Illustration of Central region, which is defined as a cylinder-shape region with 200 µm height to the *x*-direction (− 100 µm < *x* < 100 µm) and 200 µm diameter to the *y*-direction (− 100 µm < *y* = *r* < 100 µm). Time variation of the number of ions outflowing from Central region (/5 ns) estimated from (**b**) right-hand side and (**c**) left-hand side of Eq. (1). (**d**) Energy outflows due to fluid plasma motion from Central region estimated from the 2D profiles of internal energy density *e*_int_, pressure *P*, and ***v***_flow_ at *t* = 10 ns.

Here we focus on the plasma inflows because we finally found that such inflows play an important role to improve the CE as will be presented in rest of the text. Now that *n*_i_ and ***v***_flow_ were observed, it becomes possible to calculate 2D-profiles of ion flux (*n*_i_ ***v***_flow_) based on the ***v***_flow_ profiles (Fig. 3) and *n*_i_ profiles (Supplementary Figs. 3). Note that we assumed axial-symmetry of ion flux (*n*_i_ ***v***_flow_) along the *x*-axis (the laser-beam propagation axis). Based on the 2D-*n*_i_ ***v***_flow_ profiles, we estimated time variation of the number of ions outflowing from a central region. Here after we define “Central region” as a cylinder-shape region located at − 100 µm < *x* < 100 µm and − 100 µm < *y* = *r* < 100 µm as depicted in Fig. 5a. Note that “Central region” is important to discuss the total amount of EUV light emission because the EUV emission mainly comes from Central region as shown in Fig. 2e–g. In addition, the optimum ranges of *n*_e_ (3 × 10^24^–10^25^ m^-3^) and *T*_e_ (25–40 eV) for EUV sources^7,17,18^ was realized in this region, as shown in Supplementary Figs. 3d–o. The time variation of the number of ions outflowing from Central region were estimated using a right-hand side of a following integrated ion-mass conservation equation:

1 $$   \mathop{{\int\!\!\!\!\!\int\!\!\!\!\!\int}\mkern-31.2mu \bigodot}\nolimits_{v} {\frac{{\partial n_{i} }}{{\partial t}}dV =  - \mathop{{\int\!\!\!\!\!\int}\mkern-21mu \bigcirc}\nolimits_{s} {\left( {n_{i} \varvec{v}_{{flow}} } \right) \cdot d\varvec{S}} }    $$

where d*V* and d***S*** are a volume element and a surface element vectors of Central region, respectively. The regions of the volume integral *V* and the surface integral *S* are defined as the volume and the surface of Central region, respectively. Figure 5b shows the decrease of the number of ions due to the outflows from Central region during the time of 5 ns for the three different plasmas at *t* = 10 ns. As shown in Fig. 5b, in all the three cases, the Sn ions outflew from Central region, i.e., total amounts of Sn ions in Central regions decreased at *t* = 10 ns. However, due to the existence of the plasma flows toward the plasma central axis (*x*-axis), there exist the plasma inflows in Central region from the side of the cylinder (a part of the cylinder perpendicular to the *y*-axis) for the cases of the the 2.0 µs- and 2.5 µs- plasmas. As the results, the number of the ions outflowing from Central region were suppressed. To verify and do cross-check the results in Fig. 5b, the left-hand side of Eq. (1) was also calculated using the 2D-*n*_i_ profiles measured at *t* = 10 ns and 15 ns [only the 2D-*n*_i_ profile at *t* = 10 ns is shown in Supplementary Fig. 3m–o]. Figure 5c shows the results. As shown in Fig. 5b and c, the numbers of the ions outflowing from Central region during the time duration of 5 ns, which were obtained from the left-hand side [Fig. 5b] and the right-hand side [Fig. 5c] of Eq. (1), are consistent each other within the experimental error. These results indicate that the estimation of the number of ions outflowing from Central region based on the 2D-*n*_i_ ***v***_flow_ profiles are correct.

Next, we estimated internal energy loss rate (*P*_out_) in Central region due to the outflow of the plasma particles.* P*_out_ is defined as;

2 $$ P_{{out}} ~ = \mathop{{\int\!\!\!\!\!\int}\mkern-21mu \bigcirc}\nolimits_{S} {\left[ {(\rho e_{t}  + p)\user2{v}_{{flow}} } \right] \cdot d\user2{S}} $$

where *ρ* is mass density (kg/m^3^) and *e*_t_ is specific energy density (J/kg) defined as;

3 $$ \begin{array}{*{20}c} {e_{t} = e_{int} + \frac{1}{2}\left| {{\varvec{v}}_{flow} } \right|^{2} } \\ \end{array} $$

where *e*_int_ is internal energy density (J/kg) and is assumed to be $\frac{3}{2}\frac{p}{\rho }$ in this study. This estimation was performed based on the 2D profiles of *n*_e_, *T*_e_, *T*_i_, $\overline{Z}$, *p*, and ***v***_flow_ measured at *t* = 10 ns (*n*_e_, *T*_e_, *T*_i_, and $\overline{Z}$ profiles are shown in Supplementary Figs. 3). Figures 5d shows *P*_out_ with a unit of (100 mJ/10 ns). The values of *P*_out_ are all positive, which mean that the internal energy in Central region decayed at *t* = 10 ns in all cases. *P*_out_ was the highest in the 1.3 µs-plasma and the lowest in the 2.5 µs-plasma. Taking the decrease of the ions in Central region shown in Fig. 5b and c into account, this result is reasonable, i.e., *P*_out_ of the 2.0 µs- and the 2.5 µs plasmas were suppressed due to the plasma inflows.

**Figure 6:**
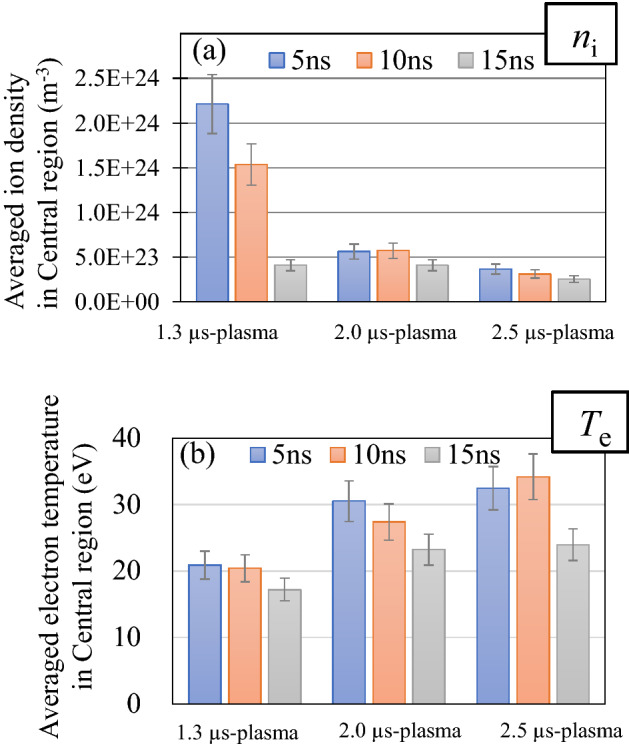
temporal evolutions of averaged (**a**) *n*_i_ and (**b**) *T*_e_ in Central region of the three plasmas.

Figures 5 show that the plasma inflows reduce outflows of both the ion number and the internal energy in Central region. It is predicted that this effect contributes to maintaining higher *n*_i_ and *T*_e_ at Central region, in which the highest EUV light emission was observed as shown in Fig. 2e–g. To confirm it, temporal evolutions of averaged *n*_i_ and *T*_e_ over Central region were calculated and shown in Fig. 6a and b. The averaged *n*_i_ and *T*_e_ were calculated from the 2D profiles of *T*_e_ and *n*_i_ measured at *t* = 5, 10, and 15 ns, which are partially shown in Supplementary Fig. 3d–f and 3m–o. As shown in Fig. 6a, the averaged *n*_i_ in Central region slowly decreased for the case of the 2.0 µs- and the 2.5 µs- plasmas, in which the plasma inflow toward Central region exist. On the other hand, for the case of the 1.3 µs- plasma, more than 60% of ions flew out from Central region for the 10 ns time duration. As for the averaged *T*_e_ in Fig. 6b, the appropriate *T*_e_ for EUV emission (25 < *T*_e_ < 40) was maintained in the 2.0 µs- and the 2.5 µs- plasmas at *t* = 5 to 10 ns. On the other hand, in the 1.3 µs- plasma, the averaged *T*_e_ was not achieved to 25 eV. These results indicate that the existence of the plasma inflows contributes to maintaining higher *n*_i_ and *T*_e_ at Central region.

**Figure 7:**
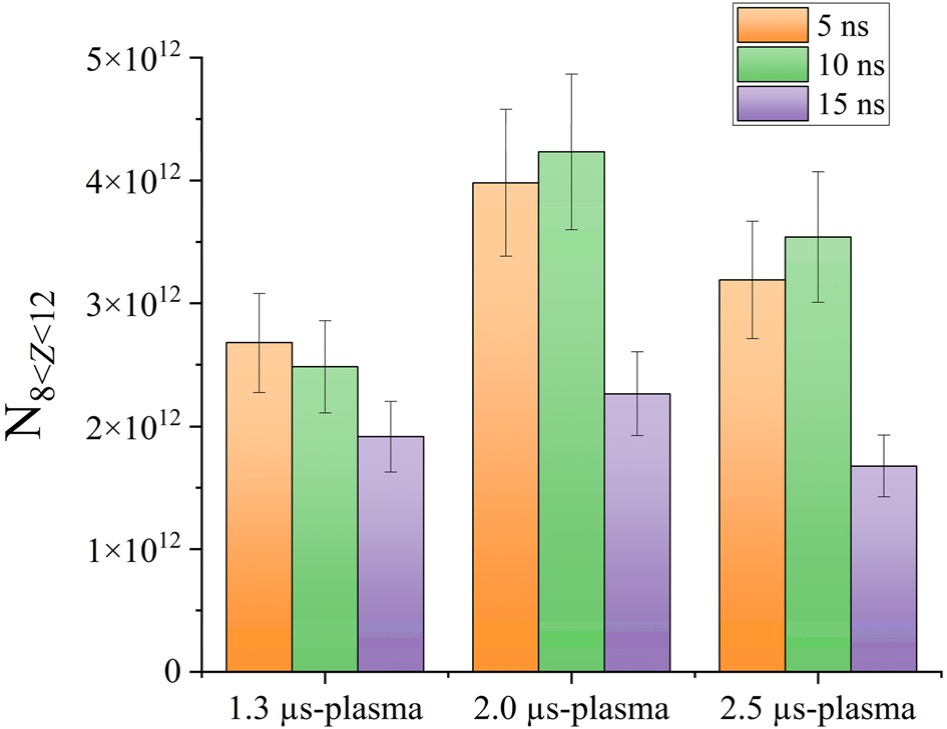
temporal evolutions of *N*_8≤Z≤12_ at Central region of the three plasmas.

To increase the total EUV emission, it is necessary to produce a larger number of Sn ions whose charge state condition is optimum (i.e., in the range of 8–12)^32^. To estimate the number of the ions in the optimum charge state condition, we count the number of ions, whose averaged charge state ($\overline{Z}$) is in the range of 8 < $\overline{Z}$ < 12 in Central region, as* N*_8≤Z≤12_. The estimation was based on the 2D profiles of *n*_i_ and $\overline{Z}$ measured at *t* = 5, 10, and 15 ns assuming that the plasmas are axial symmetry [2D profiles of *n*_i_ and $\overline{Z}$ are partially shown in Supplementary Fig. 3j–o]. As shown in Fig. 7, N8_≤Z≤12_ in the 2.0 µs-plasma is larger than that in the 1.3 µs-plasmas, although the averaged *n*_i_ of the 1.3 µs-plasma is much larger than that of 2.0 µs-plasmas [see Fig. 6a]. These results indicate that the Sn ions with the adequate *Z* were effectively produced in the 2.0 µs plasma for the existence of the plasma inflow.

**Figure 8:**
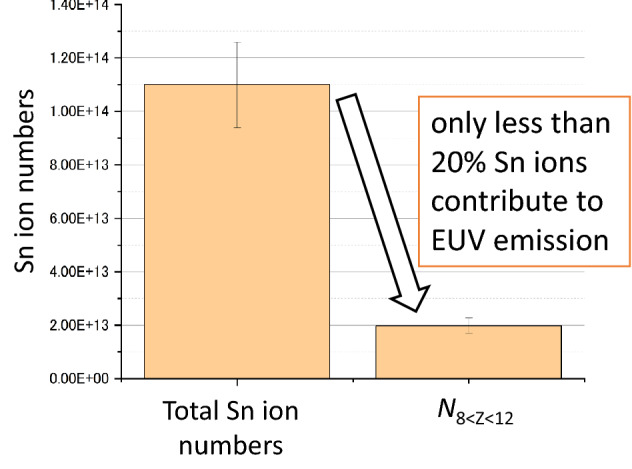
comparison of total Sn ion number and total *N*_8≤Z≤12_ in the 700 µm diameter sphere for the case of the 2.0 µs plasma at *t* = 10 ns.

The time-resolved 2D-profiles of *N*_8≤Z≤12_ and *n*_i_, both of which were revealed for the first time, suggest that there is a significant potential to increase EUV output in the future. Figure 8 shows the total number of tin ions and *N*_8≤Z≤12_ in a sphere with a diameter of 700 µm with the plasma center [(*x*, *y*, *z*) = (0, 0, 0)] as the origin. The value of *N*_8≤Z≤12_ in the 700 µm diameter sphere was analyzed using the 2D-profiles of *n*_i_ and *Z* shown in Supplementary Fig. 3 assuming the axial symmetry of the plasma along the *x*-axis. Note that the 700 µm diameter sphere is inside of the permitted etendue in EUV lithography systems^1^. Figure 8 shows that the total *N*_8≤Z≤12_ (1.9 × 10^13^) is less than 20% of the total tin ion number in the sphere with 700 µm diameter (1.1 × 10^14^). It should be mentioned that more than 90% of *N*_8≤Z≤12_ is localized within the sphere with a diameter of 300 µm with the plasma center as the origin. Therefore, almost all Sn ions produced at 300 µm < φ < 700 µm contribute little to the EUV output and there still is potential to increase EUV output power.

In conclusion, we firstly clarified time-resolved two-dimensional (2D) flow-velocity field (*v*_flow_), electron temperature (*T*_e_), electron density (*n*_e_), averaged charge state ($\overline{Z}$), and ion density (*n*_i_). These results made it possible to evaluate the time variation of the numbers of the ions outflowing from Central region and the internal energy decay due to the plasma motion in Central region (It should be mentioned that radiation losses are the main cause of the internal energy decay. Here we only evaluated the internal energy decay due to the plasma motion).

As the results, following new insights were obtained.

1. Inside the EUV light source plasmas produced with “double-pulse method”, there were the plasma inflows toward the plasma central axis (the laser propagating axis, *x*-axis). In addition, the plasma inflows were controlled by the irradiation interval time between the two lasers (the pre-pulse and the main CO_2_ lasers).
2. The plasma inflows maintain the EUV source at a temperature suitable for EUV light emission (25 eV < *T*_e_ < 40 eV) for a relatively long time (> 10 ns) and at a high ion density, i.e., The plasma inflows play an important role in improving the total EUV light emission.
3. The numbers of ions in optimum *Z* (8 < $\overline{Z}$ < 12) for EUV radiation (*N*_8≤Z≤12_) clearly changed by controlling the interval between the two laser beams. Since *N*_8≤Z≤12_ directly contributes to the total EUV output power, counting *N*_8≤Z≤12_ is crucial for improving the EUV light sources.

In addition, the CTS results suggests a bright future for improving EUV output power, i.e., there is still potential to increase the EUV output power.

## Method

### Collective Thomson scattering (CTS)

#### General remarks

**Figure 9:**
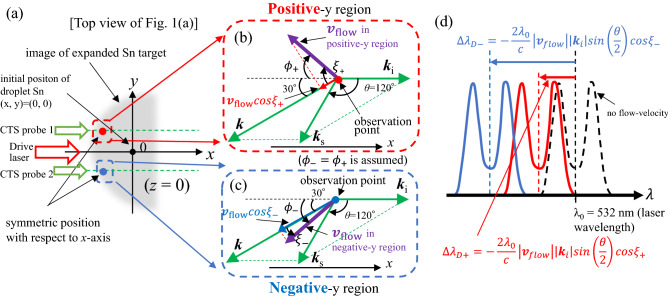
(**a**) Explanation of negative-y and positive-y regions using top view of Fig. 1a. (**b**), (**c**) Vector diagram at positive-y and negative-y regions, respectively. (**d**) Image of spectral shift due to Doppler shift at positive-y and negative-y regions.

Here, the principle of the CTS is briefly described^33,34^. The predicted Thomson scattering spectra from the EUV light source plasmas are in the collective regime when a visible probe laser is used, i.e., the scattering parameter α is larger than 1 [α = (*k*λ_D_)^−1^], where λ_D_ is the Debye length, and *k* is the absolute value of the differential scattering vector defined as ***k*** = ***k***_s_ − ***k***_i_; ***k***_i_ and ***k***_s_ are the wavevectors of the incident probe laser and the scattered light, respectively [see diagram in Fig. 9b]. The Thomson scattering spectrum in this regime comprises both an electron and an ion component^35,36^. Considering the strong background radiation from the plasma, we focused on only the ion component, for which we expected large signal-to-noise ratios against the background radiation even for a small probe-laser energy to avoid plasma heating^37,38^. The ion component spectrum reflects the ion acoustic wave frequency ω_ac_ = *k* [α^2^ / (1 + α) (*ZκT*_e_ + 3*κT*_i_) / *m*_i_)]^1/2^. The spectrum exhibits two peaks (i.e., ion features with a dip between them). The wavelength separation 2Δλ_peak_ of the two peaks is related to the probe laser wavelength λ_0_ and ω_ac_ by Δλ_peak_ = λ_0_^2^ω_ac_/(2πc), where *c* is the speed of light. $\overline{Z}$ *T*_e_ and *T*_i_ are obtained from the width Δλ_peak_ and the spectral shape, which is characterized by ion acoustic wave damping^33,34^. In addition, *n*_e_ is determined by an absolute calibration of the CTS system because the scattered light intensity is proportional to the electron density. All the plasma parameters (i.e., *T*_e_, *n*_e_, and $\overline{Z}$) are then determined assuming *T*_e_ = *T*_i_. In addition, the doppler shift of the CTS spectra gives us information of plasma flow velocity field (***v***_flow_).

#### The technical difficulties of ion component measurements for EUV source plasmas

The CTS was applied to various laser-produced plasmas (LPPs)^39–43^. However, a special challenge for the EUV light source plasmas is the very small wavelength separation of the ion features of ~ 100 pm at λ_0_ = 532 nm, which also means that the ion component is very close to the probe laser wavelength λ_0_ (i.e., 50 pm) [see Supplementary Fig. 1b and c]. Therefore, very high spectral resolution and stray light reduction are essential. Triple grating spectrometers are widely used for collective and noncollective Thomson scattering^38–45^. However, they block a wavelength range of approximately 1 nm at λ_0_ to reduce stray light (i.e., the ion component in our application is also blocked). Therefore, we built a custom spectrometer^20^. This spectrometer consists of six gratings. Four gratings are used for stray light reduction, while the other two gratings are utilized for wavelength dispersion. Thus, a spectral resolution of 12 pm and a sufficient stray-light rejection with a very narrow wavelength block range [within ± 14 pm from λ_0_ (= 532 nm)] were achieved, and the ion components from the Sn plasmas for the LPP-EUV light sources were clearly observed^19^.

#### Two-dimensional plasma flow-velocity field (${\varvec{v}}_{{{\text{flow}}}}$) measurements

**Figure 10:**
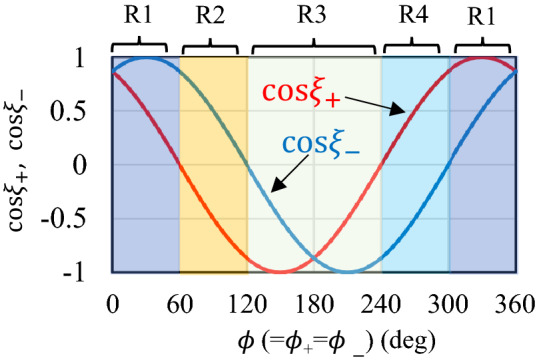
Relations among ${\text{cos}}\xi_{ + }$, ${\text{cos}}\xi_{ - }$, and $\phi { }$(= $\phi$_+_ = $\phi -$).

Here we explain in detail the way to determine two-dimensional plasma flow velocity field (${\varvec{v}}_{{{\text{flow}}}}$) profiles in our experiment (Note that there exists other way to obtain ${\varvec{v}}_{{{\text{flow}}}}$ using TS spectra^46^). First, we assume axial symmetry of ${\varvec{v}}_{{{\text{flow}}}}$ along the *x*-axis, which is same as the laser beam path. This assumption may be reasonable because all the experimental configuration and the measurements results including the expanded Sn shadowgraph profiles and in-band EUV energy profiles are axial symmetry^19^. To determine ${\varvec{v}}_{{{\text{flow}}}}$, the CTS spectra should be measured at both the positive-*y* and the negative-*y* regions [see Fig. 9a]. In addition, the measurements should be performed at the symmetric position along the *x*-axis (ex. *y* =  ± 100 µm). We used the Doppler shift of the CTS spectra measured at both the positive-*y* and the negative-*y* regions. Because the shift reflects ${\varvec{v}}_{{{\text{flow}}}}$ toward ***k***, when a direction of ${\varvec{v}}_{{{\text{flow}}}}$ is same as that of ***k***, the relationships among ***k***_i_, ${\varvec{v}}_{{{\text{flow}}}}$, and Δλ_D_***k***_, which is a wavelength shift of CTS spectrum due to Doppler shift, are as follows:

4 $$ \begin{array}{*{20}c} {\Delta \lambda_{{D_{{\varvec{k}}} }} = - \frac{{2\lambda_{0} }}{c}\left| {{\varvec{v}}_{{{\text{flow}}}} } \right|\left| {{\varvec{k}}_{i} } \right|sin\left( {\frac{\theta }{2}} \right)} \\ \end{array} $$

where *θ* is the angle between the probe laser and scattering directions, which was fixed to 120 degrees in the experiment as depicted in Figs. 2a and 9b and c. In many cases, the directions of ${\varvec{v}}_{{{\text{flow}}}}$ would be different from that of ***k***, then the expected spectral shift width would be smaller than Δλ_D_***k***_ and is written as $\Delta \lambda_{D - }$ in the negative-*y* region and as $\Delta \lambda_{D + }$ in the positive-*y* region as follows:

5 $$ \begin{array}{*{20}c} {\Delta \lambda_{D - } = \Delta \lambda_{{D_{k} }} \cos \xi_{ - } = - \Delta \lambda_{{D_{k} }} \cos \left( {\phi_{ - } - 30^{^\circ } } \right)} \\ \end{array} $$

6 $$ \begin{array}{*{20}c} {\Delta \lambda_{D + } = \Delta \lambda_{{D_{k} }} \cos \xi_{ + } = - \Delta \lambda_{{D_{k} }} \cos \left( {\phi_{ + } + 30^{^\circ } } \right)} \\ \end{array} $$

where $\xi_{ - }$ and $\xi_{ + }$ are defined as angles from ***k*** to ${\varvec{v}}_{{{\text{flow}}}}$ in the negative- and the positive-*y* regions respectively, as shown in Fig. 9b and c. In the same manner, both $\phi_{ - }$ and $\phi_{ + }$ indicate angles from $- {\varvec{k}}_{i}$ to ${\varvec{v}}_{{{\text{flow}}}}$ [See Fig. 9b and c]**.** Since ${\varvec{v}}_{{{\text{flow}}}}$ is assumed to be axial symmetry, values of $\phi_{ - }$ and $\phi_{ + }$ are same each other for the axial symmetric position along the *x*-axis. On the other hand, since the direction of ***k*** is 30 degrees different from that of $- {\varvec{k}}_{i}$, the values of $\xi_{ - }$ and $\xi_{ + }$ are different each other at the axial symmetric positions except for the case when the direction of ${\varvec{v}}_{{{\text{flow}}}}$ is parallel to ${\varvec{k}}_{i}$. Consequently, Doppler shift widths of the ion component spectra obtained at the negative-*y* region and the positive-*y* region become different each other, as depicted in Fig. 9d. In Fig. 10, ${\text{cos}}\xi_{ - }$ and ${\text{cos}}\xi_{ + }$ are plotted as the functions of $\phi \left( { = \phi_{ - } = \phi_{ + } } \right)$. As shown in the Eqs. (5) and (6), ${\text{cos}}\xi_{ - }$ and ${\text{cos}}\xi_{ + }$ are phase-shifted to − 30 degrees and + 30 degrees from cos $\phi$, respectively.

From the experimental results, plus/minus signs of $\Delta \lambda_{D - }$ and $\Delta \lambda_{D + }$ are determined. Using combinations of the signs of $\Delta \lambda_{D - }$ and $\Delta \lambda_{D + }$, four regions (R1-R4) can be classified as shown in Fig. 10 and Table 1 (R1–R4 are explained in Table 1). After determining the region, $\phi \left( { = \phi_{ - } = \phi_{ + } } \right)$ is obtained by dividing (5) by (6), or dividing (6) by (5):

7 $$ \begin{array}{*{20}c} {\frac{{\Delta \lambda_{D - } }}{{\Delta \lambda_{D + } }} = \frac{{{\text{cos}}\left( {\phi_{ - } + 30^{^\circ } } \right)}}{{{\text{cos}}\left( {\phi_{ + } - 30^{^\circ } } \right)}}} \\ \end{array} $$

**Table 1:** Definition of four regions (R1–R4) and corresponding ranges of $\phi { }$(= $\phi +$ = $\phi -$).

Region	R1	R2	R3	R4
$\Delta \lambda_{D + }$	Negative	Positive	Positive	Negative
$\Delta \lambda_{D - }$	Negative	Negative	Positive	Positive
$\phi$(= $\phi +$ = $\phi -$)	$0^{ \circ } \le \phi < 60^{ \circ } ,300^{ \circ } \le \phi < 360^{ \circ }$	$60^{ \circ } \le \phi < 120^{ \circ }$	$120^{ \circ } \le \phi < 240^{ \circ }$	$240^{ \circ } \le \phi < 300^{ \circ }$

Once $\phi \left( { = \phi_{ - } = \phi_{ + } } \right)$ is obtained, $\left| {{\varvec{v}}_{{{\text{flow}}}} } \right|$ is fixed using (4). Consequently, ${\varvec{v}}_{{{\text{flow}}}}$ is determined.

#### Examples of $\Delta \lambda_{D + }$ and $\Delta \lambda_{D - }$ profiles

**Figure 11:**
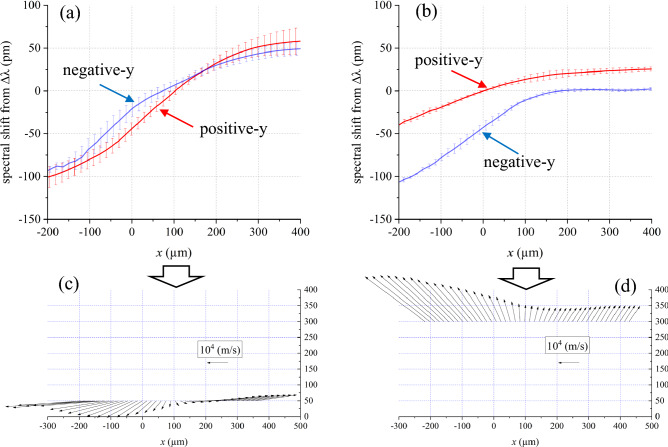
One-dimensional $\Delta \lambda_{D + }$ and $\Delta \lambda_{D - }$ profiles measured at (**a**) *y* =  ± 50 μm (the 2.0 µs-plasma, *t* = 10 ns) and (**b**) *y* =  ± 300 μm (the 1.3 µs-plasma, *t* = 10 ns). (**c**), (**d**): one-dimensional ***v***_flow_ profiles calculated from $\Delta \lambda_{D + }$ and $\Delta \lambda_{D - }$ profiles in (**a**) and (**b**), respectively.

Figure 11a and b are one-dimensional $\Delta \lambda_{D + }$ and $\Delta \lambda_{D - }$ profiles measured at (a) *y* =  ± 50 μm (the 2.0 µs-plasma, *t* = 10 ns) and (b) *y* =  ± 300 μm (the 1.3 µs-plasma, *t* = 10 ns). As shown in these figures, the $\Delta \lambda_{D - } {\text{and}} \Delta \lambda_{D + }$ profiles are clearly different when the measurement condition was changed. The error bars show standard deviations of the measurements. The classification using Table 1 and the calculation using (7) give us $\phi_{ - } \left( { = \phi_{ + } } \right)$. Then, $\left| {{\varvec{v}}_{{{\text{flow}}}} } \right|$ is determine using (5) or (6), and (4). Finally, the 1D profiles of ${\varvec{v}}_{{{\text{flow}}}}$ are obtained as show in Fig. 11c and d. We estimated the measurement uncertainties of the velocity angle and the velocity magnitude are typically ± 15° and ± 15%, respectively. The error range of the velocity angle is mainly determined by the values of $\Delta \lambda_{D + } /\Delta \lambda_{D - }$ or $\Delta \lambda_{D - } /\Delta \lambda_{D + }$, which are related with values of $\phi { }$(= $\phi$_+_ = $\phi -$) as shown in Fig. 10 and Table 1. For example, at *x* = 0 in Fig. 11a, the value of $\Delta \lambda_{D - } /\Delta \lambda_{D + }$ is 0.48 ± 0.18. This value corresponds to $\phi$ = 329° ± 11°. The uncertainty of the magnitude of *v*_flow_ is mainly determined by the standard deviations of $\Delta \lambda_{D + }$ or $\Delta \lambda_{D - }$. When both absolute values of $\Delta \lambda_{D - }$ and $\Delta \lambda_{D + }$ are smaller than 10 pm, it is difficult to determine ***v***_flow_ because of measurement uncertainties.

## Supplementary Information

Supplementary Information.

## Data Availability

The datasets used and/or analyzed during the current study available from the corresponding author on reasonable request.
