# Supplementary material for: Observation of plasma inflows in laser-produced Sn plasma and their contribution to extreme-ultraviolet light output enhancement
Source: Sci Rep. 2023 Feb 1;13:1825. doi: 10.1038/s41598-023-28500-8 (PMC9892586; doi:10.1038/s41598-023-28500-8)
Supplement: Supplementary file 1 — Supplementary Information. [file 41598_2023_28500_MOESM1_ESM.pptx]

## Slide 1
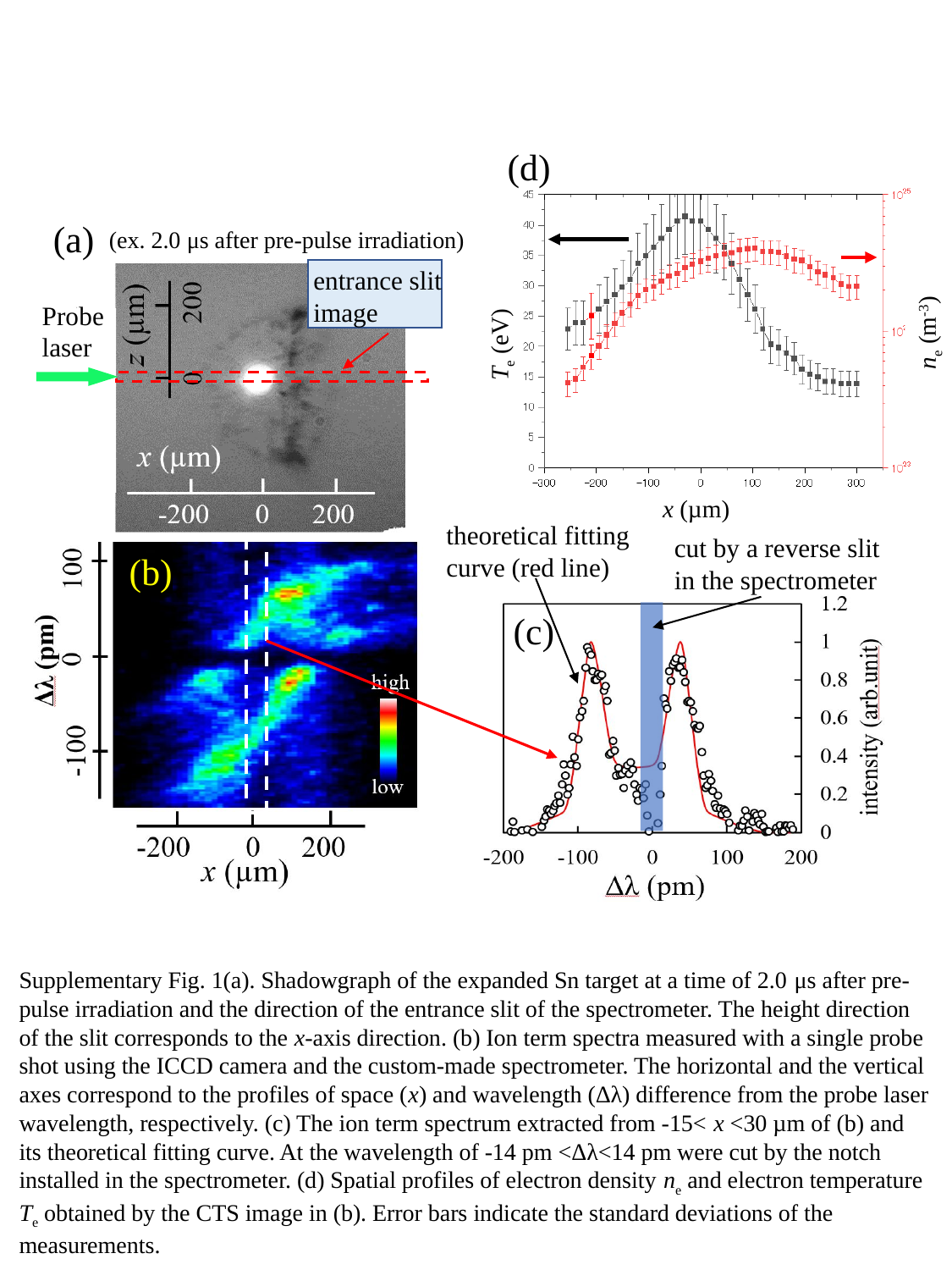

(d)
(a)
 (ex. 2.0 μs after pre-pulse irradiation)
entrance slit image
Probe laser
ne (m-3)
Te (eV)
x (µm)
theoretical fitting curve (red line)
cut by a reverse slit in the spectrometer
(b)
(c)
Supplementary Fig. 1(a). Shadowgraph of the expanded Sn target at a time of 2.0 μs after pre-pulse irradiation and the direction of the entrance slit of the spectrometer. The height direction of the slit corresponds to the x-axis direction. (b) Ion term spectra measured with a single probe shot using the ICCD camera and the custom-made spectrometer. The horizontal and the vertical axes correspond to the profiles of space (x) and wavelength (Δλ) difference from the probe laser wavelength, respectively. (c) The ion term spectrum extracted from -15< x <30 µm of (b) and its theoretical fitting curve. At the wavelength of -14 pm <Δλ<14 pm were cut by the notch installed in the spectrometer. (d) Spatial profiles of electron density ne and electron temperature Te obtained by the CTS image in (b). Error bars indicate the standard deviations of the measurements.

## Slide 2
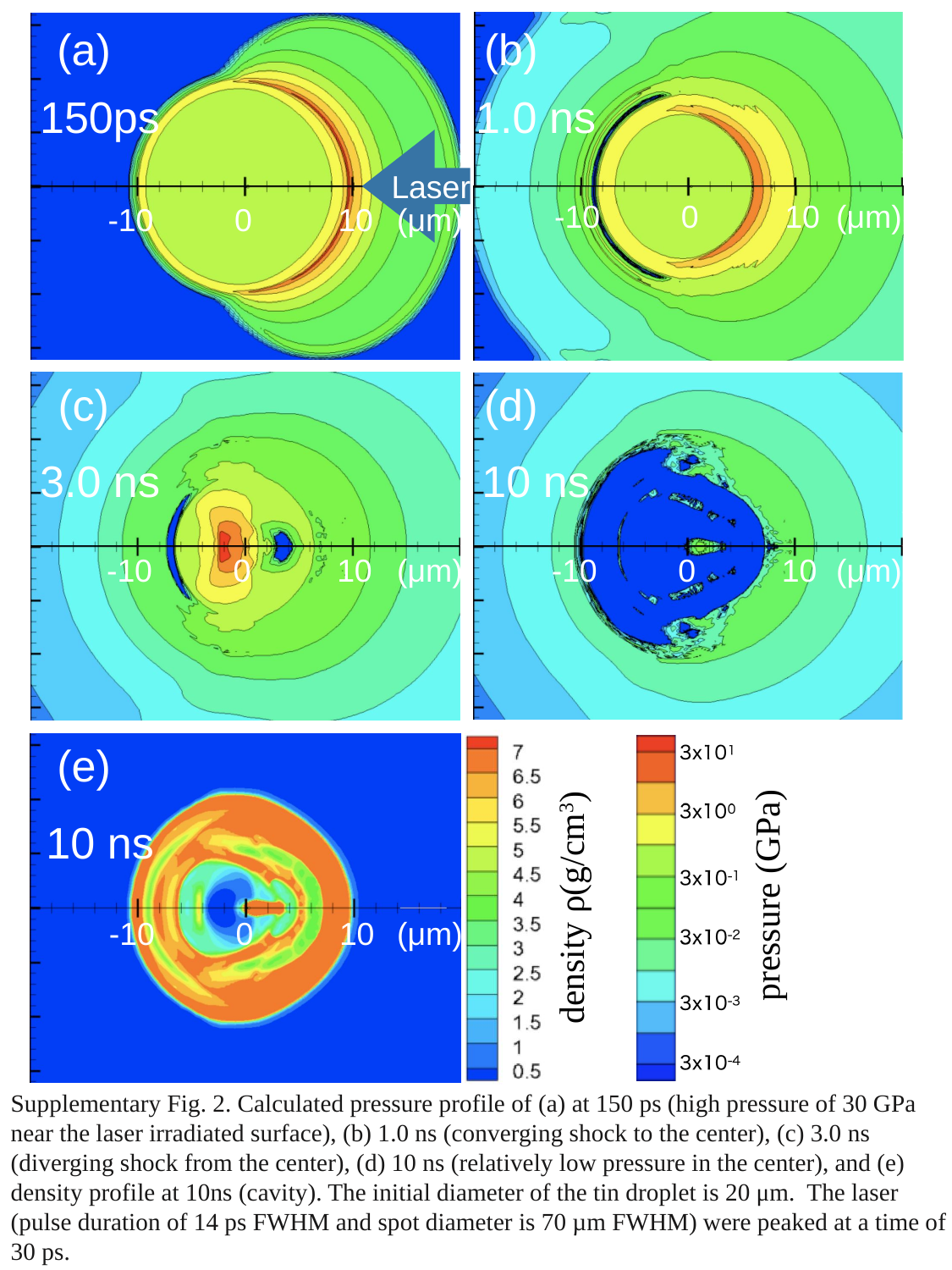

(a)
 (b)
150ps
1.0 ns
Laser
 (c)
 (d)
3.0 ns
10 ns
 (e)
10 ns
pressure (GPa)
density ρ(g/cm3)
-10
0
10
(μm)
-10
0
10
(μm)
-10
0
10
(μm)
-10
0
10
(μm)
-10
0
10
(μm)
Supplementary Fig. 2. Calculated pressure profile of (a) at 150 ps (high pressure of 30 GPa near the laser irradiated surface), (b) 1.0 ns (converging shock to the center), (c) 3.0 ns (diverging shock from the center), (d) 10 ns (relatively low pressure in the center), and (e) density profile at 10ns (cavity). The initial diameter of the tin droplet is 20 μm. The laser (pulse duration of 14 ps FWHM and spot diameter is 70 µm FWHM) were peaked at a time of 30 ps.

## Slide 3
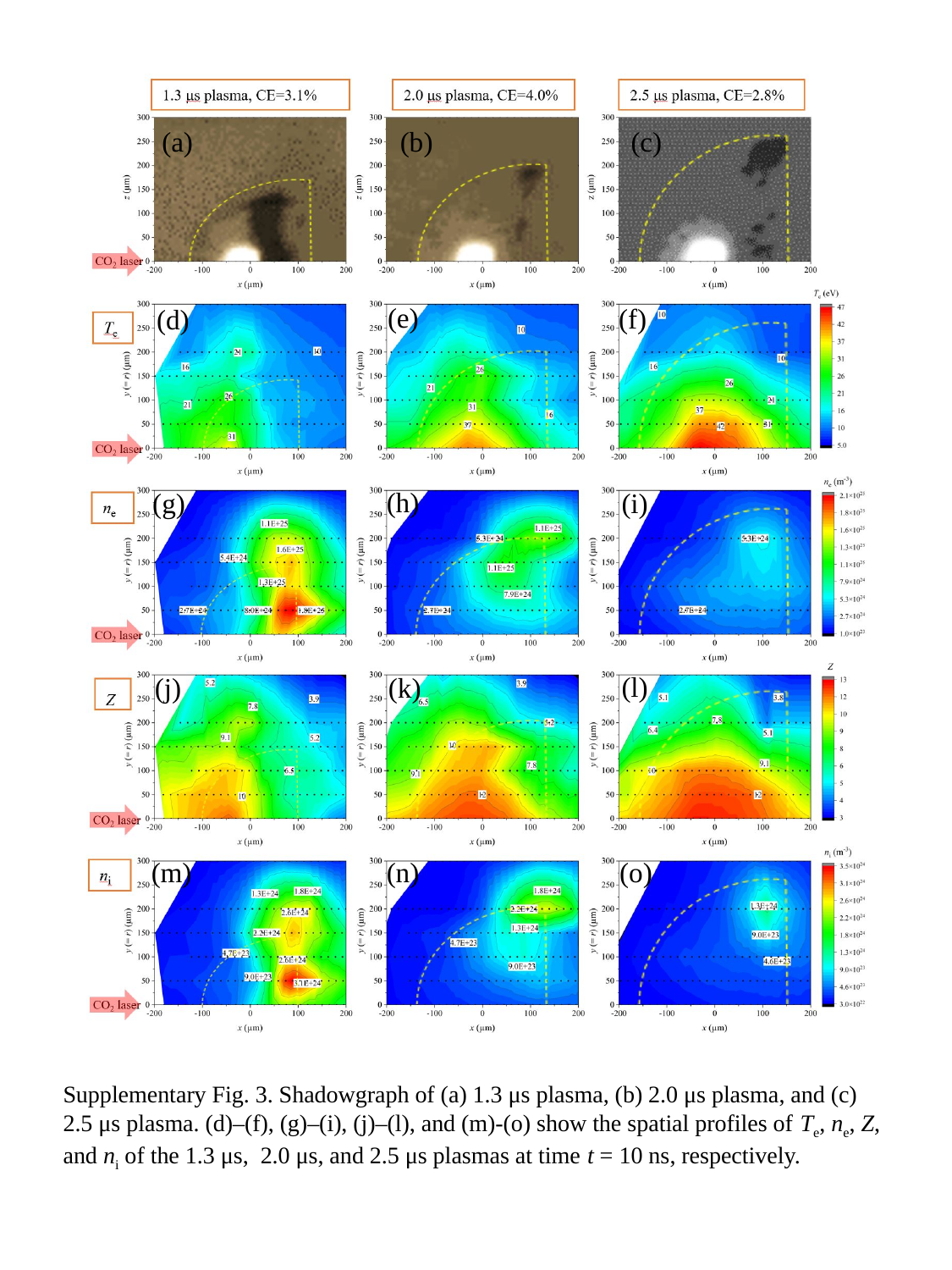

(a)
(b)
(c)
(e)
(f)
(d)
(h)
(g)
(i)
(l)
(j)
(k)
(m)
(n)
(o)
Supplementary Fig. 3. Shadowgraph of (a) 1.3 μs plasma, (b) 2.0 μs plasma, and (c) 2.5 μs plasma. (d)–(f), (g)–(i), (j)–(l), and (m)-(o) show the spatial profiles of Te, ne, Z, and ni of the 1.3 μs, 2.0 μs, and 2.5 μs plasmas at time t = 10 ns, respectively.
